# Long-lasting adverse effects of short-term stress during the suckling–mastication transition period on masticatory function and intraoral sensation in rats

**DOI:** 10.1007/s10266-023-00887-w

**Published:** 2024-01-10

**Authors:** Ayano Katagiri, Masaharu Yamada, Hajime Sato, Hiroki Toyoda, Hitoshi Niwa, Takafumi Kato

**Affiliations:** 1 Department of Oral Physiology, Osaka University Graduate School of Dentistry, 1-8 Yamadaoka, Suita-shi, Osaka 565-0871 Japan; 2 Department of Dental Anesthesiology, Osaka University Graduate School of Dentistry, 1-8 Yamadaoka, Suita-shi, Osaka 565-0871 Japan; 3 Division of Pharmacology, Meikai University School of Dentistry, 1-1 Keyakidai, Sakado-shi, Saitama 350-0283 Japan

**Keywords:** Hypoxic stress, Intraoral hypersensitivity, Masticatory function, Maternal separation, Suckling-mastication transition period

## Abstract

Early-life stress affects brain development, eventually resulting in adverse behavioral and physical health consequences in adulthood. The present study assessed the hypothesis that short-term early-life stress during infancy before weaning, a period for the maturation of mastication and sleep, poses long-lasting adverse effects on masticatory function and intraoral sensations later in life.

Rat pups were exposed to either maternal separation (MS) or intermittent hypoxia (IH-Infancy) for 6 h/day in the light/sleep phase from postnatal day (P)17 to P20 to generate “neglect” and “pediatric obstructive sleep apnea” models, respectively. The remaining rats were exposed to IH during P45–P48 (IH-Adult). Masticatory ability was evaluated based on the rats’ ability to chew pellets and bite pasta throughout the growth period (P21–P70). Intraoral chemical and mechanical sensitivities were assessed using two-bottle preference drinking tests, and hind paw pain thresholds were measured in adulthood (after P60).

No differences were found in body weight, grip force, and hind paw sensitivity in MS, IH-Infancy, and IH-Adult rats compared with naïve rats. Masticatory ability was lower in MS and IH-Infancy rats from P28 to P70 than in naïve rats. MS and IH-Infancy rats exhibited intraoral hypersensitivity to capsaicin and mechanical stimulations in adulthood. The IH-Adult rats did not display inferior masticatory ability or intraoral hypersensitivity.

In conclusion, short-term early-life stress during the suckling–mastication transition period potentially causes a persistent decrease in masticatory ability and intraoral hypersensitivity in adulthood. The period is a “critical window” for the maturation of oral motor and sensory functions.

**Supplementary Information:**

The online version contains supplementary material available at 10.1007/s10266-023-00887-w.

## Introduction

The Developmental Origins of Health and Disease theory suggests that maternal conditions and other environmental factors during the early developmental period (“critical window”) contribute to later-life disease susceptibility [1]. Exposure to maltreatment, such as abuse and neglect, during childhood potentially results in long-term adverse behavioral and physical health outcomes in adulthood [2–6]. As the first 2 weeks of life in rodents have been recognized as the critical window for the development of motor and sensory functions and behaviors [7, 8], preclinical studies examining the effects of early-life stress on development have typically been designed to apply stress conditions during the first 2 postnatal weeks in rodents [9]. In humans, the prenatal period and first 3 postnatal years are critical developmental periods for biological and behavioral maturation and possess high levels of opportunity for and vulnerability to maltreatment [2, 10]. However, rodents are altricial species that undergo considerable postnatal development. In rats, two weeks after birth is equivalent to the human fetal stage [11]. Therefore, the effects of childhood maltreatment on nervous system development have not been studied extensively in most preclinical studies. A few studies have indicated that early-life stress exposure after postnatal day (P)15 produces permanent microglial sensitization and neuronal death in the brain that persists into adulthood [12]. This suggests that early-life stress after the first 2 postnatal weeks potentially impacts the development of specific functions, as not all physical and behavioral functions are entirely developed before the first 2 weeks of age in rodents.

Pronounced postnatal development involves mastication, an indispensable oromotor function for nutrient intake. Mastication is characterized by a transition from suckling the mother’s milk to masticating solid food. Infancy is a crucial period for acquiring complex masticatory ability in humans and mammals [13, 14]. Masticatory function is acquired through various factors, such as molar eruption (after P17), central nervous system maturation, oral sensory inputs emergence, and motor learning [13–18]. Behavioral studies have reported that infant rats commence food intake at approximately P17–P18 [19, 20]. Thus, P17–P20, before weaning on P21, is a critical window for the acquaintance of masticatory function in rodents.

Notably, this period corresponds with sleep maturation [21, 22]. Pediatric obstructive sleep apnea (OSA), which is characterized by repetitive episodes of intermittent hypoxia (IH) during sleep, has become widely recognized; 1.2–5.7% of children may have OSA [23]. Pediatric OSA increases the risk of developing neuropsychiatric disorders, such as attention-deficit hyperactivity disorder (ADHD) [24]. This indicates that IH in the early life of vulnerable individuals can ultimately lead to long-lasting alterations in neural function.

Therefore, we assessed the hypothesis that early-life stress, with maternal separation (MS) and IH as “neglect” and “pediatric OSA” models, respectively, from P17 to P20 before weaning exerts long-lasting adverse effects on later-life masticatory function and intraoral sensation.

## Materials and methods

**Fig. 1:**
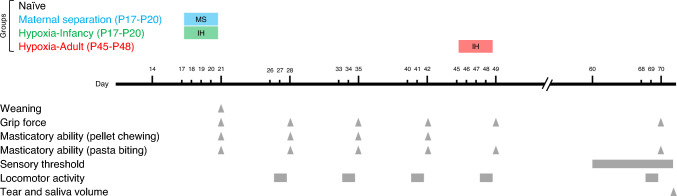
Experimental design. Body weight and grip force were measured on P21, P28, P35, P42, P49, and P70. Pellet-chewing tests were performed at P21, P28, P35, and P42. Pasta-biting tests were performed on P21, P28, P35, P42, P49, P56, and P70. Sensory thresholds of the intraoral, ocular, and hind paw areas were measured in adulthood after P60. Locomotor activity was measured for 24 h on P26 (starting at 15:00, light phase)–P27 (finishing at 15:00, dark phase), P33–P34, P40–P41, P47–P48, P54–P55, and P68–P69. Tear and saliva volumes were measured on the day all behavioral tests were completed after P71. IH intermittent hypoxia, MS maternal separation

The Animal Experiments Committee approved the protocol of this animal study, which was conducted at the Osaka University Graduate School of Dentistry (R-04–010). All experimental procedures were performed in accordance with the ARRIVE guidelines 2.0 (Animal Research: Reporting of In Vivo Experiments) [25]. Sixty male Sprague–Dawley rats (Japan SLC, Shizuoka, Japan) were evaluated in this study. Rat litters consisted of 6–9 pups. Data of three rats were excluded from the analysis because they could not habituate to the behavioral test environment. The rats were housed in a light-controlled environment and climate (dark/light period 03:00–15:00/15:00–03:00; each day started at 03:00; temperature: 23 ± 0.5 °C). The pups were weaned at 08:00 on P21 (Fig. 1). Food and water were provided ad libitum. The animals were randomly allocated to each treatment group. All efforts were made to reduce the number of animals used in the experiment.

### Experimental procedures

The experimental design is illustrated in Fig. 1. Rats were divided into four groups: 1) a naïve group, 2) a group exposed to MS for 4 days during P17–P20 (MS group), 3) a group exposed to IH for 4 days during P17–P20 (IH-Infancy group), and 4) a group exposed to IH during P45–P48 (IH-Adult group). All behavioral tests, excluding the recording of locomotor activity, were performed between 11:00 and 15:00 in the dark/wake periods by a well-trained investigator blinded to the models. Body weight and grip force were measured before fasting on P21, P28, P35, P42, P49, and P70. Pellet-chewing tests were performed at P21, P28, P35, and P42. Pasta-biting tests were performed on P21, P28, P35, P42, P49, P56, and P70. Sensory thresholds of the intraoral, ocular, and hind paw areas were measured in adulthood after P60. Locomotor activity was measured for 24 h on P26 (starting at 15:00, light phase) –P27 (finishing at 15:00, dark phase), P33–P34, P40–P41, P47–P48, P54–P55, and P68–P69. Tear and saliva volumes were measured on the day all behavioral tests were completed after P71.

### Neglect and OSA models

The pups allow to freely access food and water during 6 h of MS and IH exposure. Mothers were left undisturbed in their home cage during the separation procedure. At the end of the MS and IH (infancy) protocol, pups were returned to their home cage.

### *Neglect model (Maternal separation)*

From P17 to P20, the pups were separated from their mothers for 6 h/day during the light/sleep period from 15:00 to 21:00. Cages containing rat pups were placed on a warm plate (38 °C) during MS.

### *OSA model (intermittent hypoxia)*

Infant (separated from mothers) and adult rats were placed in a tightly sealed Plexiglas chamber (W 25 × D 41 × H 17 cm^3^) during P17–P20 (IH-Infancy) or P45–P48 (IH-Adult) for 6 h/day in the light/sleep period from 15:00 to 21:00. Cages containing rat pups were placed on a warm plate (38 °C) during IH for infant rat. The IH protocol consisted of cycles of hypoxia reaching 5% O_2_ at the nadir mixed with N_2_ for 3 min, followed by 3 min of normoxia. N_2_ was delivered to the chamber at a rate of 7.0–8.8 L/min. The compressed air was delivered at a rate of approximately 21 L/min. The gas flushing into the chamber was automatically switched from compressed air to N_2_ and subsequently back to compressed air (SEVENz Planning Inc., Tokyo, Japan). The IH protocol has been described previously [26].

### Grip force test

Each rat was held by its tail and passed over a wire mesh grid connected to a strain gauge [27]. The maximum forelimb grip force during the three-to-four trials at 10-min intervals was determined.

### Locomotor activity

A Nano-Tag® device (18.8 × 14.2 × 7.1 mm^3^, 2.7 g; Kissei Comtec Co., Ltd., Nagano, Japan) was implanted under the back skin of each rat under 3% isofluorane anesthesia on P21 after weaning. The Nano-Tag® device was switched on > 2 h before the commencement of recording [29], and the data were percutaneously transferred to the Nano-Tag® Viewer program (Kissei Comtec Co., Ltd.) after recording using a FeliCa reader (RC-S360; Sony Corp., Tokyo, Japan) under light isoflurane anesthesia. Locomotor activity was recorded every 30 s and stored on the Nano-Tag® device. The data were represented as the average value for each hour. For measurements involving the Nano-tag® device, activity was defined as cross-count data, providing a count of the number of times the XYZ acceleration vector-synthesized waveform crossed the threshold levels from the bottom (170/min) to the top (170/min) per recording interval. Locomotor activity was measured in group-housed rats (two to three rats/cage) in each experimental group to prevent social-separation stress.

### Masticatory functions (pellet-chewing and pasta-biting tests)

The rats were individually habituated to the experimental chamber in a Plexiglas chamber, fasted for 3 h prior to testing [28], and allowed to drink water freely. The amounts of pellet (MF#4 certified rat diet, diameter: 3.2 mm, length: 10.0–20.0 mm; Oriental Yeast Co., Ltd., Tokyo, Japan) chewing for 10 s and pasta (diameter: 1.2 mm, length: 2.0–8.0 mm) biting for 30 s were measured 5 times and calculate the average of 3 times excluding the maximum and minimum values. The pellet-chewing test was conducted until P42 and was limited by pellet size.

### Sensory-threshold measurements

### *Eyeblink reflex (capsaicin and mechanical stimulation) *

Rats were habituated to a Plexiglas chamber for 1 h prior to testing. Eyeblinks were counted for 3 min after capsaicin (1.0 µM, 15.0 µL) stimulation or for 20 s after mechanical (0.04 g von Frey filament) stimulation of the ocular surface. Eyes were rinsed with saline immediately after capsaicin instillation. Mechanical stimulation was applied three times at intervals of > 30 min, and the average was calculated. Eyeblinks included partial and complete lid closures. The details have been described previously [26].

### *Intraoral sensitivity (capsaicin and mechanical stimulation)*

The rats were deprived of water for 22 h, including 1 h of pre-testing habituation in the Plexiglas chamber. The two-bottle preference drinking test was administered for 2 h. Rats were allowed free access to two adjacent bottles. Three types of stimulations were used for the two-bottle preference drinking test: vehicle-0.33 µM capsaicin, vehicle-1.0 µM capsaicin, and spout with/without mechanical stimulation. The bottles with spouts with/without mechanical stimulation contained distilled water. The spout (diameter: 6.0 mm) of the bottle subjected to mechanical stimulation was made of optical fibers. The optical fibers (diameter: 0.5 mm) were arranged in parallel around the spout without any spaces in between, and the tip of each optic fiber was randomly set at 2.0–3.0 mm from the edge of the spout. Each experiment was performed on two successive days. On 2 consecutive days, the positions of two bottles (vehicle-0.33 µM capsaicin, vehicle-1.0 µM capsaicin, and spout with/without mechanical stimulation) were reversed each day to avoid positional preference. Each bottle was weighed before and after the 2-h drinking test session to measure the volume of fluid consumed. Consumption of 0.33 µM capsaicin, 1.0 µM capsaicin, and distilled water in bottle with mechanical stimulation spout was quantified as the percentage of the total volume consumed during the 2-h drinking test sessions on each test day for each rat. The average ratio of 2 consecutive days was calculated. The rats received water ad libitum in their home cages during non-drinking-test periods. The details of the two-bottle preference drinking test have been described previously [26].

### *Hind paw sensitivity*

The rats were habituated to a Plexiglas chamber with a wire-mesh floor for approximately 15 min until major grooming activity ceased. Mechanical sensitivity was assessed using calibrated von Frey filaments (2, 5, 8, 10, 15, 20, and 25 g; cutoff: 25 g) applied to the mid-plantar left hind paw to avoid the footpads. The withdrawal threshold for hind paw mechanical stimulation was defined as the minimum pressure required to evoke at least three escapes in five trials separated by 1-min intervals. A positive response was recorded when the paw was sharply withdrawn. Flinching immediately after the removal of the von Frey filament was also considered a positive response.

### Tear and saliva volume measurement

Measurements of spontaneous tear and saliva volumes were performed for 2 min by increasing the wet length of the phenol red thread (Zone-Quick™, Ayumi Pharmaceutical Co., Tokyo, Japan) after fasting and water deprivation for 3 h under pentobarbital sodium anesthesia (80 mg/kg, intraperitoneal) before perfusion. The average tear volumes in bilateral eyes were calculated. For saliva-volume measurements, a phenol red thread (Zone-Quick™) was gently placed in the sublingual area. Except for the tip, the thread was covered with a polyethylene tube (SP45, length: 3.0 cm, Natsume Seisakusho, Tokyo, Japan) to avoid contact with the mucosa and lower lip. The details has been described previously [30, 31].

### Statistical analysis

The Kruskal–Wallis test, followed by the Dunn test, was used to analyze and compare threshold, tear volume, and saliva volume measurements in each group. Two-way analysis of variance, followed by the Bonferroni test, was used for analysis and comparison at each time point to assess group differences and baseline directly (day 1 of each experiment) in body weight, grip force, pellet chewing, pasta biting, and locomotor activity (Prism version 7.02, GraphPad Software). The data are presented as the mean ± standard error of the mean. Statistical significance was set at *p* < 0.05. A sample size of five per treatment group was calculated to provide 80% power at p < 0.05. The actual numbers and p-values in each graph are summarized in the Supplemental Tables.

## Results

### General conditions

**Fig. 2:**
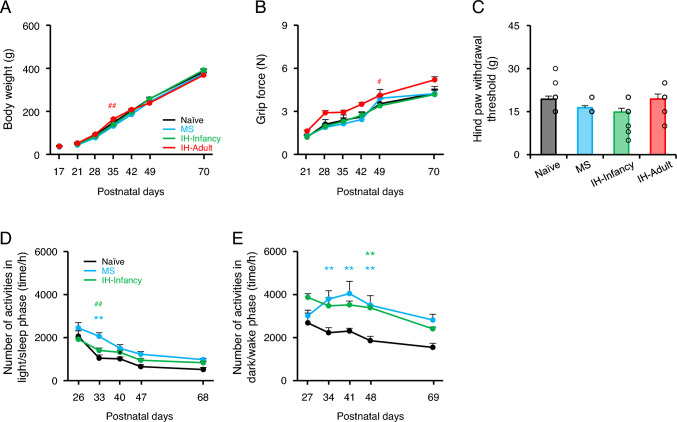
General condition and sensitivity of hind paw. **A** Body weight. ##p < 0.01: MS vs. IH-Adult. **B** Grip force. #p < 0.05: IH-Infancy vs. IH-Adult. **C** Hind paw withdrawal threshold. **D** Number of activities in the light/sleep phase per hour. **p < 0.01: vs. Naïve. ##p < 0.01: MS vs. IH-Infancy. **E** Number of activities in the dark/wake phase per hour. **p < 0.01: vs. Naïve. IH-Adult intermittent hypoxia-adulthood (P45–P48), IH-Infancy intermittent hypoxia infancy (P17–P20), MS maternal separation (P17–P20)

No significant differences were noted in body weight and grip force in MS, IH-Infancy, and IH-Adult rats compared with naïve rats. Body weight and grip force increased significantly with age in each group (Figs. 2A–B and Supplemental Tables; naive, n = 9; MS, n = 6; IH-Infancy, n = 9; IH-Adult, n = 5). MS, IH-Infancy, and IH-Adult rats did not exhibit any significant differences in hind paw withdrawal thresholds compared with naïve rats (Fig. 2C; naive, n = 15; MS, n = 11; IH-Infancy, n = 13; IH-Adult, n = 8). These results suggest that MS and hypoxic stress during the suckling-mastication transition period as well as 4 days of hypoxia in adulthood did not influence body-weight gain, muscle-strength growth, or the body pain threshold of the limbs.

The number of locomotor activities during the light/sleep and dark/wake phases gradually decreased with growth. Interestingly, the number of activities in the dark/wake phase was significantly higher in MS and IH-Infancy rats than in naïve rats (Fig. 2D–E and Supplemental Tables; naïve, n = 5; MS, n = 5; IH-Infancy, n = 5). These results suggest that MS and IH during the suckling-mastication transition period induces hyperlocomotor activity.

### Underdevelopment of masticatory function

**Fig. 3:**
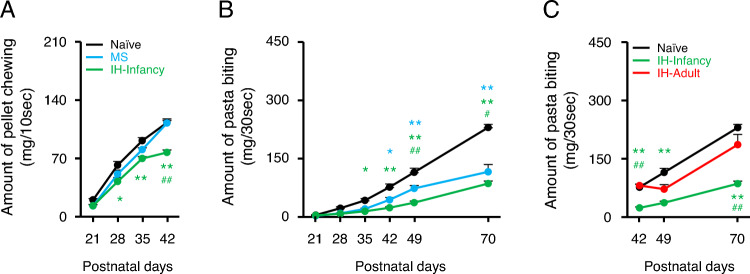
Pellet-chewing and pasta-biting ability per unit time. **A** Amount of pellet chewing per 10 s. *p < 0.05, **p < 0.01: vs. Naïve. ##p < 0.01: MS vs. IH-Infancy. **B** Amount of pasta biting per 30 s. *p < 0.05, **p < 0.01: vs. Naïve. #p < 0.05, ##p < 0.01: MS vs. IH-Infancy. **C** Amount of pasta biting per 30 s. **p < 0.01: vs. Naïve. ##p < 0.01: IH-Infancy vs. IH-Adult. IH-Adult intermittent hypoxia-adulthood (P45–P48), IH-Infancy intermittent hypoxia infancy (P17–P20), MS maternal separation (P17–P20)

Pasta-biting (incision) and pellet-chewing tests were conducted to evaluate masticatory ability because the masticatory sequence starts with food preparation and incision followed by chewing. Incision and chewing use different central neural system regions to generate rhythmic jaw movement [13, 32]. The amount of pellet chewing and pasta biting significantly increased with development in all the groups (Fig. 3A and Supplemental Tables; naive, n = 14; MS, n = 8; IH-Infancy, n = 9. Figure 3B; naïve, n = 8; MS, n = 8; IH-Infancy, n = 9). IH-Infancy rats exhibited a lower amount of pellet chewing than naïve and MS rats (Fig. 3A). The amount of pasta biting was significantly lower in both MS and IH-Infancy rats than in naïve rats (Fig. 3B and Supplemental Tables). To confirm the effect of IH during development, adult rats were subjected to IH for 4 days from P45 to P48 in the IH-Adult group. No significant difference was observed between IH-Adult and naïve rats throughout the experimental period (Fig. 3C and Supplemental Tables; IH-Adult, n = 8), and IH-Infancy rats demonstrated less pasta biting than IH-Adult rats. These results suggest that MS and hypoxic stress during the suckling-mastication transition period, but not during adulthood, induce the underdevelopment of masticatory function.

### No effect on ocular sensitivity in adulthood

**Fig. 4:**
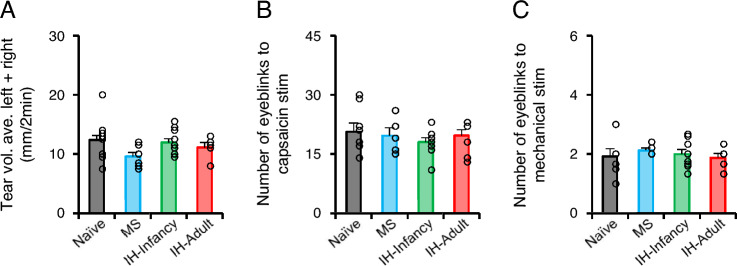
Tear volume and sensitivity of the eye to capsaicin and mechanical stimulation. **A** Average spontaneous tear volume of the left and right eyes. **B** Number of eyeblinks evoked by 1.0 μM capsaicin administration to the ocular surface for 3 min. **C** Number of eyeblinks evoked by mechanical stimulation (0.04 g von Frey filament) to the ocular surface for 20 s. IH-Adult intermittent hypoxia-adulthood (P45–P48), IH-Infancy, intermittent hypoxia infancy (P17–P20), MS maternal separation (P17–P20)

No significant differences in spontaneous tear volume were noted among all rat groups (Fig. 4A; naive, n = 15; MS, n = 8; IH-Infancy, n = 11; IH-Adult, n = 5). Furthermore, no significant differences in the number of eyeblinks in response to capsaicin and mechanical stimulations were observed among all rat groups (Fig. 4B–C; naive, n = 8; MS, n = 5; IH-Infancy, n = 9; IH adult, n = 8). These results suggest that MS and hypoxic stress during the suckling-mastication transition period did not affect ocular sensitivity.

### Hypersensitivity to capsaicin and mechanical stimulation of the tongue in adulthood

**Fig. 5:**
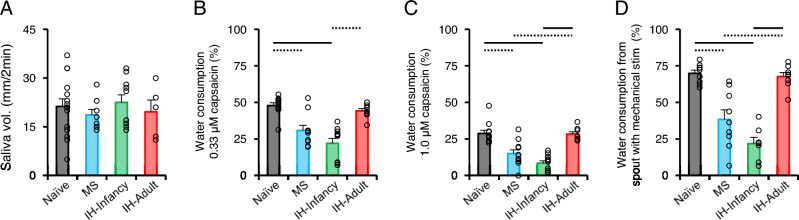
Saliva volume and sensitivity of the tongue to capsaicin and mechanical stimulation. **A** Spontaneous saliva volume. **B** Water consumption of 0.33 μM capsaicin solution for 2 h. Dotted line: p < 0.05, black line: p < 0.01. **C** Water consumption of 1.0 μM capsaicin solution for 2 h. Dotted line: p < 0.05, black line: p < 0.01. **D** Water consumption from a spout with mechanical stimulation for 2 h. Dotted line: p < 0.05, black line: p < 0.01 IH-Adult, intermittent hypoxia-adulthood (P45–P48); IH-Infancy intermittent hypoxia infancy (P17–P20), MS maternal separation (P17–P20)

No significant differences in spontaneous saliva volume were observed among all rat groups (Fig. 5A; naive, n = 15; MS, n = 8; IH-Infancy, n = 10; IH-Adult, n = 5). Consumption of capsaicin solution and distilled water from a spout with mechanical stimuli was significantly lower in MS and IH-Infancy rats than in naïve and IH-Adult rats (Fig. 5B; Naive, n = 11; MS, n = 10; IH-Infancy, n = 11; IH-Adult, n = 8; Fig. 5C; Naive, n = 11; MS, n = 11; IH-Infancy, n = 11; IH-Adult, n = 8; Fig. 5D; Naive, n = 9; MS, n = 9; IH-Infancy, n = 7; IH-Adult, n = 8). Contrastingly, IH in adulthood did not affect the consumption of capsaicin solution or distilled water from a spout with a mechanical stimulus. There was no significant difference in the total volume consumed during 2 h of the drinking test sessions among all four groups (data not shown). These results suggest that intraoral hypersensitivity induced by early-life stress was not due to mouth dryness [30].

## Discussion

Present findings revealed that short-term early-life stress during a period for maturation of mastication and sleep results in the underdevelopment of masticatory function, intraoral hypersensitivity, and behavioral abnormality in adulthood. Additionally, IH had a stronger effect than MS on masticatory ability and intraoral sensory development.MS and IH, as experimental stressors, disrupt normal developmental processes in pups. Previous studies have demonstrated that MS [33] and IH [34] before P15 do not affect body weight increases and feeding behaviors in adulthood. Herein, body weight and grip force, which can estimate physical strength, did not differ among naïve, MS, and IH-Infancy groups. This suggests that early-life stress during P17–P20 does not affect body growth, daily food intake, and the development of physical strength in later life. Additional assessments of ocular and hind paw sensitivity further support that short-term MS and IH during P17–P20 did not cause aversive effects in the extra-oral areas of the body in adulthood.

Nonetheless, MS and IH during P17–P20, but not in IH-Adults, resulted in lower masticatory ability in adulthood. The masticatory ability to consume the test food was assessed based on pellet chewing and pasta biting, in which the jaw-closing muscles (i.e., masseter) play significant roles in biting, crushing, and grinding solid foods [13]. In addition, IH in adulthood did not alter masticatory function. Therefore, the present study clearly corroborates the hypothesis that early-life stress during the transition from suckling to mastication has long-lasting adverse effects on masticatory function later in life. Several factors should also be considered. In rodents, mastication behaviors emerge after P17 [13, 15, 19, 20] in association with neurochemical and anatomical alterations in the trigeminal motor system. In jaw-closing motoneurons, N-methyl-D-aspartate receptors significantly increase [35–37], and phenotypic changes in inhibitory synapses from gamma-aminobutyric acid to glycine occur approximately on postnatal 3–4 weeks [38]. Furthermore, the first molars erupt and start to occlude between P17 and P18 [16], suggesting that the periodontal afferent feedback loop in molar chewing may mature during this period. Therefore, these changes in the nervous system are susceptible to MS and IH as they influence neuroplastic changes [39, 40]. Second, site-specific and time-specific susceptibility to IH is present in the skeletal muscles. Respiratory, limb, and geniohyoid (suckling) muscles, but not masseter muscles, are vulnerable to gestational IH in adolescent rodents [41, 42]. The initial signs of the alpha motor endplates are found in the masseter muscle at P18 [43], followed by rapid growth in masseter muscle fibers [44]. Therefore, IH during P17–P20 potentially leads to reduced masticatory muscles growth. Third, tactile stimulation with the mother maintains the secretion of thyroid and growth hormones [45, 46]. Deficiency of these hormones in MS and IH-Infancy can decrease the number of large masseter motoneurons and delay masticatory function [45].

This study yielded remarkable additional findings. First, MS and IH between P17–P20, as opposed to between P45–48, led to intraoral hypersensitivity. Notably, our previous study revealed that IH for 8–16 days in adulthood resulted in transient intraoral hypersensitivity to capsaicin, which disappeared after IH had ceased [26]. These findings underscore the importance of the timing of stress exposure, as MS and IH during infancy have long-lasting effects on the intraoral sensory system. Second, MS and IH during infancy induced mechanical allodynia in the oral structure but not in the cornea or hind paw in adulthood. Previous studies have indicated that the sensory and motor systems for exploratory behavior develop before mastication [8]. Eye opening, walking, and running typically commence at P15 [8] before the emergence of mastication and eruption of molars (i.e., P17) [16, 19, 20]. Differences in the timing of development among the eyes, limbs, and oral structures can be correlated with the critical window of pain sensation. The sensitive period for long-term alterations in nociceptive responses is reportedly limited to the first 2 weeks of neonatal life in rats [47]. MS during P2–P15 increased pain sensitivity in the hind paws of adult rodent offspring [4]. The maturation of these nociceptive pathways lasts more than 3 weeks after birth [48–50]. Interestingly, desensitization of C-fibers by subcutaneous capsaicin treatment at birth leads to the loss of pain perception in adulthood, while desensitization after P14 does not change pain thresholds [51, 52]. In addition, the descending pain facilitation pathway exerts a powerful excitatory influence on spinal nociception until P21, after which the inhibitory pathways begin to drive [49, 53]. This descending excitation in early life potentially contributes to the activity-dependent development of nociceptive pathways [7]. Considering the above information, the present study suggests that MS and IH during P17–P20 may alter the development of nociceptive sensory pathways. However, the critical window of nociceptive thresholds in the cornea and limbs, which precedes that in the intraoral structures, may contribute to the time-dependent development of the motor system.

As discussed above, decreased masticatory ability and intraoral hypersensitivity in adulthood were induced independently after MS and IH during infancy in this study. However, decreased masticatory ability is possibly associated with intraoral hypersensitivity, as orofacial pain potentially attenuates masticatory performance by decelerating rhythm and lowering force [54]. Contrastingly, decreased masticatory ability in infancy may alter pain perception in adulthood because hard-food mastication suppresses pain by driving an opioid descending system via the trigeminal sensory pathway and somatosensory cortex [55].

Consistent with our findings (MS or IH during P17–P20), IH during P7–P11 (nadir O_2_: 10%, 6 h/day) has been found to induce hyperlocomotor activity during adulthood [56]. Contrarily, IH (nadir O_2_: 5%, 16 days) in adulthood resulted in hypolocomotor activity (data not shown). Therefore, MS stress and pediatric apnea-induced hypoxia may be a potential mechanism contributing to the pathogenesis of ADHD [24]. Our locomotor activity findings suggest that MS and IH during the suckling-mastication transition period influences not only oral function development but also neurobehavioral development in the later life.

This study has some limitations. Only behavioral assessments were made in this study; therefore, the possibilities discussed require further investigation of anatomical and neurophysiological changes. Second, this study assessed masticatory ability using pellet chewing and pasta biting over with short timeframes within each experimental period. Therefore, whether decreased masticatory ability changes feeding behavior, such as prolonged feeding, remains unknown. This should be further investigated in association with the increased locomotor activity during dark/wake period. Third, this study did not examine the morphological effects of stress, such as delayed tooth eruption. This is unlikely because stress is applied after the critical window for tooth eruption [57].

Here, MS and IH were used as “neglect” and “pediatric OSA” models, respectively. The results indicate that short-term early-life stress during infancy potentially leads to a subsequent oral dysfunction. If appropriate masticatory function is not acquired during growth period, habilitation of mastication is reportedly impossible in adulthood in rodents [58]. Neuroimaging studies in humans have revealed that brain regions associated with memory and learning are activated during mastication, and impaired masticatory function induces dementia [59]. Hence, investigating the masticatory function in children affected by early-life stress and implementing interventions to prevent future declines in masticatory ability and intraoral pain hypersensitivity is crucial.

In conclusion, the short-term early-life stress during a period for maturation of mastication and sleep (P17–P20) before weaning potentially causes a persistent decrease in masticatory ability accompanied by intraoral hypersensitivity and behavioral abnormality in adulthood.

### Supplementary Information

Below is the link to the electronic supplementary material.

Supplementary file1 (XLSX 42 KB)

## Data Availability

The datasets generated and/or analyzed during the current study are available from the corresponding author on reasonable request.

## Abbreviations

P: Postnatal day,
MS: Maternal separation
IH: Intermittent hypoxia
IH-Infancy: Intermittent hypoxia in infancy
IH-Adult: Intermittent hypoxia in adulthood
OSA: Obstructive sleep apnea
ADHD: Attention-deficit hyperactivity disorder
